# Supplementary material for: Stacking Model‐Based Classifiers for Dealing With Multiple Sets of Noisy Labels
Source: Biom J. 2025 Mar 12;67(2):e70042. doi: 10.1002/bimj.70042 (PMC11898607; doi:10.1002/bimj.70042)
Supplement: Supplementary file 1 — Supporting Information [file BIMJ-67-e70042-s001.zip › density-based-ensemble-model-main/competitors_real_data/netlab-master/netlab-master/help/netunpak.htm]

Netlab Reference Manual netunpak


# netunpak

## Purpose

Separates weights vector into weight and bias matrices.

## Synopsis

```
net = netunpak(net, w)
```

## Description

`net = netunpak(net, w)` takes an net network data structure `net` and
a weight vector `w`, and returns a network data structure identical to
the input network, except that the componenet weight matrices have all
been set to the corresponding elements of `w`. If there is
a `mask` field in the `net` data structure, then the weights in
`w` are placed in locations corresponding to non-zero entries in the
mask (so `w` should have the same length as the number of non-zero
entries in the `mask`).

## See Also

`netpak`, `netfwd`, `neterr`, `netgrad`

---

**Pages:**
Index


---

Copyright (c) Ian T Nabney (1996-9)
